# Wireless high-resolution surface facial electromyography mask for discrimination of standardized facial expressions in healthy adults

**DOI:** 10.1038/s41598-024-70205-z

**Published:** 2024-08-20

**Authors:** Paul F. Funk, Bara Levit, Chen Bar-Haim, Dvir Ben-Dov, Gerd Fabian Volk, Roland Grassme, Christoph Anders, Orlando Guntinas-Lichius, Yael Hanein

**Affiliations:** 1 Department of Otorhinolaryngology, Jena University Hospital, Friedrich-Schiller-University Jena, Am Klinikum 1, 07747 Jena, Germany; 2 School of Electrical Engineering, Tel Aviv University, Tel Aviv, Israel; 3 Tel Aviv University Center for Nanoscience and Nanotechnology, Tel Aviv University, Tel Aviv, Israel; 4 Facial-Nerve-Center Jena, Jena University Hospital, Jena, Germany; 5 Center for Rare Diseases, Jena University Hospital, Jena, Germany; 6 Division Motor Research, Pathophysiology and Biomechanics, Department of Trauma, Hand and Reconstructive Surgery, Jena University Hospital, Friedrich-Schiller-University Jena, Jena, Germany; 7 Department of Prevention, Biomechanics, German Social Accident Insurance Institution for the Foodstuffs and Catering Industry, Erfurt, Germany; 8 Sagol School of Neuroscience, Tel Aviv University, Tel Aviv, Israel; 9 X-Trodes, Herzliya, Israel

**Keywords:** Neurology, Neurological disorders, Diagnosis

## Abstract

Wired high resolution surface electromyography (sEMG) using gelled electrodes is a standard method for psycho-physiological, neurological and medical research. Despite its widespread use electrode placement is elaborative, time-consuming, and the overall experimental setting is prone to mechanical artifacts and thus offers little flexibility. Wireless and easy-to-apply technologies would facilitate more accessible examination in a realistic setting. To address this, a novel smart skin technology consisting of wireless dry 16-electrodes was tested. The soft electrode arrays were attached to the right hemiface of 37 healthy adult participants (60% female; 20 to 57 years). The participants performed three runs of a standard set of different facial expression exercises. Linear mixed-effects models utilizing the sEMG amplitudes as outcome measure were used to evaluate differences between the facial movement tasks and runs (separately for every task). The smart electrodes showed specific activation patterns for each of the exercises. 82% of the exercises could be differentiated from each other with very high precision when using the average muscle action of all electrodes. The effects were consistent during the 3 runs. Thus, it appears that wireless high-resolution sEMG analysis with smart skin technology successfully discriminates standard facial expressions in research and clinical settings.

## Introduction

Facial electromyography (EMG) is widely used in different research and clinical domains. Although alternative imaging approaches were explored in recent decades, in particular computer vision-based tools, facial EMG remains a gold standard, especially in clinical research, as it offers quantitative and muscle specific information. In psychophysiological and emotional research facial EMG is commonly used to study facial movements and emotions by measuring the activation of facial muscles associated with different facial functional and emotional expressions^1,2^. It also offers insight into the neuromuscular activities underlying speech and facial movements, aiding in the diagnosis and monitoring of neuromuscular diseases. In medical research, facial EMG is used to diagnose and assess neuromuscular disorders affecting facial muscles. In particular, in rehabilitation medicine, facial EMG serves as a valuable tool for assessing the effectiveness of treatments aimed at restoring facial muscle function following injury or in conditions such as facial palsy.

A major emphasis in neuromuscular assessments is the evaluation of functional movements such as eye closure, or lip pursing as they determine patients’ ability to regain proper functions such as blinking and speech. Functional facial movements like eye closure, pursing the lips or emotional expressions recruit the action of several facial muscles^3^. Therefore, recordings in psychological settings are usually performed on the surface of facial muscles via multi-channel surface EMG (sEMG)^4^. Recently, we have shown that high resolution facial sEMG can discriminate with high accuracy and reliability between standard facial functional movements and also between the six basic emotions^5–7^. As this approach relies on manual placement of multiple electrodes, it is very complex and time-consuming. Our particular setup required simultaneous application of 29 electrodes, in specific locations, per side of the face for each examination^4,8^.

Recently, a smart skin technology consisting of screen-printed carbon electrode array (16 electrodes, 4 mm in diameter) was developed and tested. The arrays allow a wireless recording of facial muscle activation and mapping of facial expression of emotion in a natural and settings^9–11^. The 16 multi-channel facial EMG-based system is a dry electrode array patch and self-adhesive. This allows an easy and fast sticking to the face within a few seconds.

In this investigation, we studied the suitability of these electrodes for clinical research of facial muscles. Specifically, we tested if the electrode arrays allow a reliable discrimination of important functional movements, and discussed if the results achieve an accuracy like conventional facial EMG. Emotional expressions were not the central focus of the study. By demonstrating similar discrimination with the wireless system compared to the results obtained with an elaborate positioning of many single electrode in the face we aim to demonstrate the superiority of the electrode arrays. Such wireless adhesive electrode arrays could be used for a wide range of applications in medical and psychological research.

## Materials and methods

### Healthy participants

The study included 37 healthy adult volunteers (22 female; mean age: 28 years, age range: 20 to 57 years). Participants with a neurological disease or history of botulinum toxin injection in the face, facial surgery or trauma were excluded. The experiments were conducted in accordance with relevant guidelines and regulations under approval from the Institutional Ethics Committee Review Board at Tel Aviv University (no. 0005248-2) in accordance with the Helsinki guidelines and regulations for human research. All participants gave written informed consent to participate in the study. Individuals depicted in the figures of this manuscript have provided written informed consent for their images to be published in an online open-access publication.

### Standardization of repeated facial exercises

**Figure 1:**
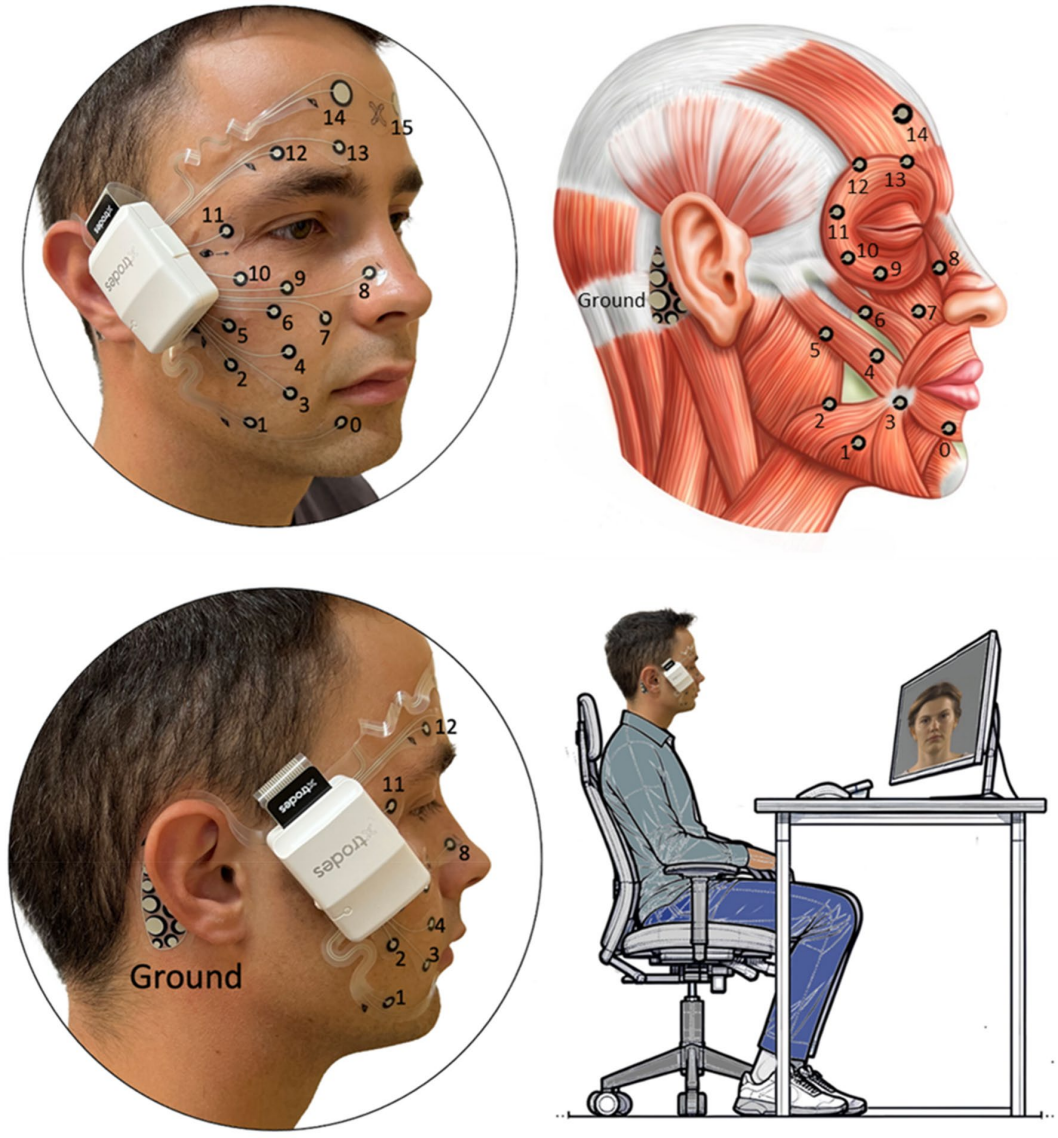
The experimental setup and the positions of the electrodes on the face: Channel 0–14 on the right side of the face and channel 15 on the contralateral left forehead. In addition, a reference electrode is placed on the right mastoid. Participants were sitting in front of a computer screen, followed the instructions of a video tutorial, and imitated the shown facial expressions (Illustration: courtesy of Sonja Burger).

Participants were seated in an upright posture in front of a computer screen which provided detailed instructions regarding the examination. The examination lasted about 25 min, started with a self-explanatory video tutorial that allows for standardized and reliable instructions for performing facial movements given by a human instructor^12,13^. Following the video instructions, the participants performed 11 facial expressions: Face at rest (no movement), wrinkling of the forehead, closing the eyes normally (gentle eye closure), closing the eyes forcefully (forceful eye closure), nose wrinkling, smiling with closed mouth, smiling with open mouth, lip puckering (pursing lips), blowing-out the cheeks (cheek blowing), snarling, and depressing the lower lip. The 11 exercises are shown in Supplementary Fig. S1. All facial movements were performed three times (run 1–3: R1, R2, R3). The schematic setup is shown in Fig. 1.

### Wireless facial surface electromyography registration

Surface electromyography (sEMG) measurement was conducted with a monopolar montage of screen-printed carbon dry disposable electrode masks (Fig. 1; XTELC0004005RM, X-trodes Inc., Herzliya, Israel). Data were recorded with a wireless data acquisition unit (DAU, X-trodes Inc., Herzliya, Israel) which attaches to the electrode mask. The DAU saved the sEMG data to a micro-SD card and transmitted a continuous Bluetooth signal to the controlling Android tablet application at the same time. The DAU supported up to 16 unipolar channels (2 μV noise root-mean-square (rms), 0.5–700 Hz) with a sampling rate of 4000 S/s, 16-bit resolution, an input range of ± 12.5 mV and input impedance of 10^7^ Ω. A 620 mAh battery supported the DAU operation for a duration of up to 16 h.

The disposable electrode masks consisted of 16 electrodes (channels). Channels 0–14 were attached to the right side of the face and channel number 15 to the left forehead (cf. Fig. 1). An internal ground electrode was placed on the right mastoid process. The scheme of the X-trodes electrodes is printed to a fixed-size polyurethane adhesive layer. The mask was attached to the skin first by peeling off the adhesive film from the center of the mask and then applying the mask while ensuring the arrow indicating the eye points towards the eye. Next, the outer protective films were peeled away one by one to fully attach the mask. The mask’s design offered considerable flexibility, allowing for adjustable positioning of the combined electrodes.

To mitigate potential technical and physiological artifacts before proceeding with any further analysis, the signals were centered and bandpass filtered within the 10 to 500 Hz range. Additionally, to account for circuit interferences, a 50 Hz notch filter was applied. sEMG amplitudes were quantified as mean rms values (single interval duration for calculation: 125 ms) during the steady state contraction phases of every facial expression and sEMG channel. The rms data for all study participants can be found in the Supplementary Data File S1. We also applied maximum normalization to the data, aiming to equalize for both individual variability and movement-dependent amplitude changes, thereby standardizing amplitude comparisons across different facial movements.

### Topographical heatmaps for visualization of the sEMG activity

To demonstrate the spatial pattern of EMG activity across the face, topographical heatmaps following the methodology of earlier work were generated^5^. In summary, this involved a modified 4-nearest neighbor interpolation of the EMG rms values with the inverse square of the distance as weight^14^. Due to the non-spherical nature of the face’s conducting surface, the weight of the most distant (fourth) electrode was progressively reduced to zero in the vicinity of the change between two fourth neighbors to avoid spatial discontinuity^15^. There are no significant asymmetries between the right and left sides of the face as healthy participants performed the 11 designated movements^5^. Data collection was uniformly conducted on the right side using the sEMG electrode mask. Therefore, to achieve a holistic depiction of the signal’s regional distribution throughout the entire face, the data were mirrored (not including electrode channel 15) onto the left side as a preliminary measure prior to interpolation.

### Statistics

A linear mixed effects model (LMM) was applied for the analysis of the activation pattern of all electrodes (channels). With such a method fixed and random effects are considered, which elevates analyses for highly complex data^16,17^. Initially, the sEMG amplitudes for all facial expressions were calculated as mean values with an estimated 95% confidence interval (CI). All electrodes were included in the calculation to evaluate the main effects of the parameters “run” (movement repetition R1, R2, R3) and “movement” (the 11 exercises) together with their interactions. “Run” and “movement” were modeled as fixed effects with a random intercept per subject. Initially, all main effects and interactions were calculated, but for the final analysis, only the significant main effects together with significant interactions remained in the calculation. Adjustment for multiple comparisons for differences between the tested facial movements was performed by the least significant difference. The significance level was set to 5%. For the heatmaps, mean values and standard deviation were calculated. Amplitude heatmaps are displayed in two ways: (i) Minimum to maximum scaling separately per facial movement for topographical pattern recognition (absolute heatmaps), (ii) Minimum to maximum scaling across all facial expressions for intensity comparison between facial movements (relative heatmaps). To allow a comparison to other data sets, heatmaps displaying the topographical distribution of the dimensionless coefficient of variation (CV) were calculated additionally.

### Ethics statement

Written informed consent was obtained from all participants. The ethics committee of the Tel Aviv University approved the study.

## Results

### Activation of the different electrodes of the wireless electrode masks during the facial movement exercises

**Figure 2:**
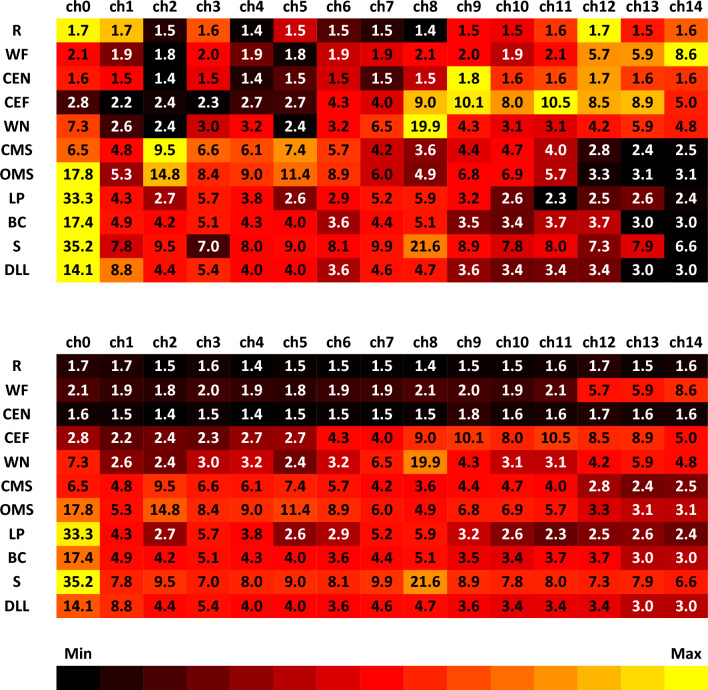
Activation of the 14 ipsilateral electrodes (ch = channel 0 to 14) during the 11 exercises. *Upper panel*: Min–Max normalization per row (i.e., per facial expression). *Lower panel*: Min–Max normalization across all facial expressions. Average sEMG amplitudes in µV is color-coded from low activation (black) to high activation (yellow). *R* at rest, *WF* wrinkling of the forehead, *CEN* closing the eyes normally, *CEF* closing the eyes forcefully, *WN* wrinkling of the nose, *CMS* closed mouth smiling, *OMS* open mouth smiling, *LP* lip puckering, *BC* blowing-out the cheeks, *S* snarling, *DLL* depressing lower lips.

Figure 2 summarizes the activation of each electrode during the following facial exercises: rest (R), wrinkling of the forehead (W), closing the eyes normally (CEN), closing the eyes forcefully (CEF), wrinkling of the nose (WN), closed mouth smiling (CMS), open mouth smiling (OMS), lip puckering (LP), blowing-out the cheeks (BC), snarling (S), and depressing lower lips (DLL). Details on the average activation of each channel (electrode) during each run and for each of the 11 exercises of all subjects are shown in the Supplementary Figs. S2–S12. The electrical activity per channel decreased (but not significantly) from run to run for most channels and most exercises. The activity did not vary much in-between the channels at rest. Differences of the activity in-between the channels were not compared statistically separately for each task. We describe the differences here only descriptively: Channels 12–14 showed pronounced activity during frowning. All channels were activated almost uniformly during gentle eye closure. This changed during forced eye closure. Here, channels 8–13 were mainly activated. Channel 8 was mainly activated during nose wrinkling, whereas the other channels were nearly silent. Smiling with a closed mouth showed a maximal activation in channel 2, and the signal continuously decreasing as the distance from channel 2 increased (with the minimal value obtained in channel 14). Channels 0 and 2 were dominant during smiling with an open mouth and the decrease in signal as the distance increased was not as clearly as for the exercise with a closed mouth. Lip puckering and blowing the cheeks led to an exclusive activation of channel 0. The muscle activation decreased over the runs for blowing the cheeks. Snarling was mainly different in the activation from blowing the cheeks by the simultaneous activation of channel 0 and channel 8. Depressing the lower lips was dominated by the activation of channels 0 and 1.

### Discrimination of the facial muscle activity during the different facial exercises by wireless sEMG recordings using the entire activation pattern of all channels

**Figure 3:**
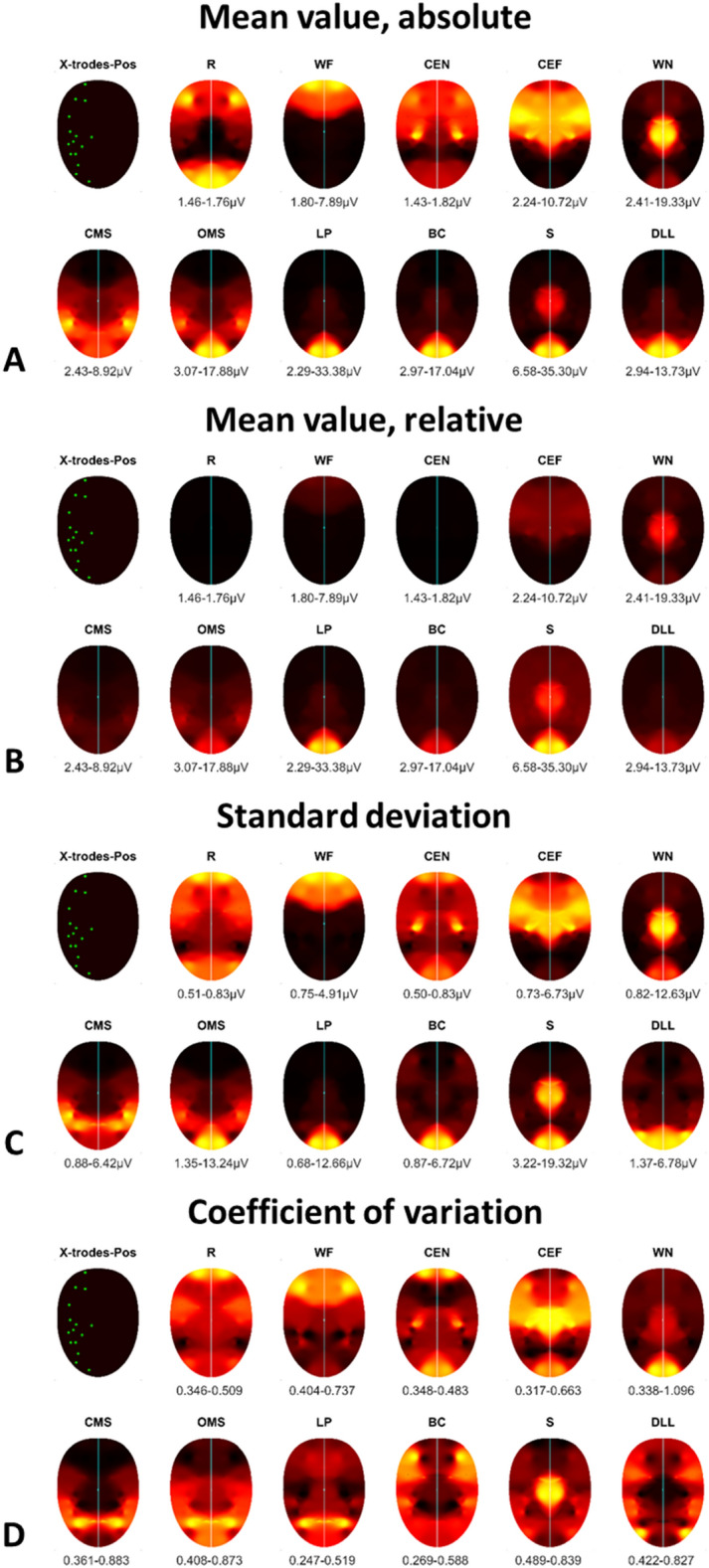
Topographical normalized heatmaps of facial muscle activation patterns during specific facial exercises. For better visualization, the measured activation on the right side was mirrored to the left side. The upper left map shows the localization of the electrodes (channels, also with their left-sided mirrors). The variability of the muscle activity in µV is plotted below each heatmap. (**A**) Absolute mean values: minimum to maximum scaling separately per facial movement for topographical pattern recognition. (**B**) Relative mean values: minimum to maximum scaling across all facial expressions for intensity comparison between facial movements. (**C**) Standard deviation of A; (**D**) coefficient of variation (CV) of A. *R* at rest, *WF* wrinkling of the forehead, *CEN* closing the eyes normally, *CEF* closing the eyes forcefully, *WN* wrinkling of the nose, *CMS* closed mouth smiling, *OMS* open mouth smiling, *LP* lip puckering, *BC* blowing-out the cheeks, *S* snarling, *DLL* depressing lower lips. The same color coding as in Fig. 2 was used.

The specific facial muscle activation patterns during the 11 exercises are visualized with topographic heatmaps in Fig. 3. The normalized heatmaps show localized activation with clear resemblance to the anticipated activation region. The results of the discrimination analysis are shown in Table 1. The average muscle activation of all three runs of facial exercises was evaluated as well for each run separately. Nearly all facial exercises could be differentiated from each other: 82% of all exercises could be differentiated by the wireless sEMG recordings when using the average data of all three runs. 73%, 81%, and 84% of the exercises could be differentiated from each other in run 1, run 2, and run 3, respectively (all had p < 0.05 after correction for multiple testing).

**Table 1:**
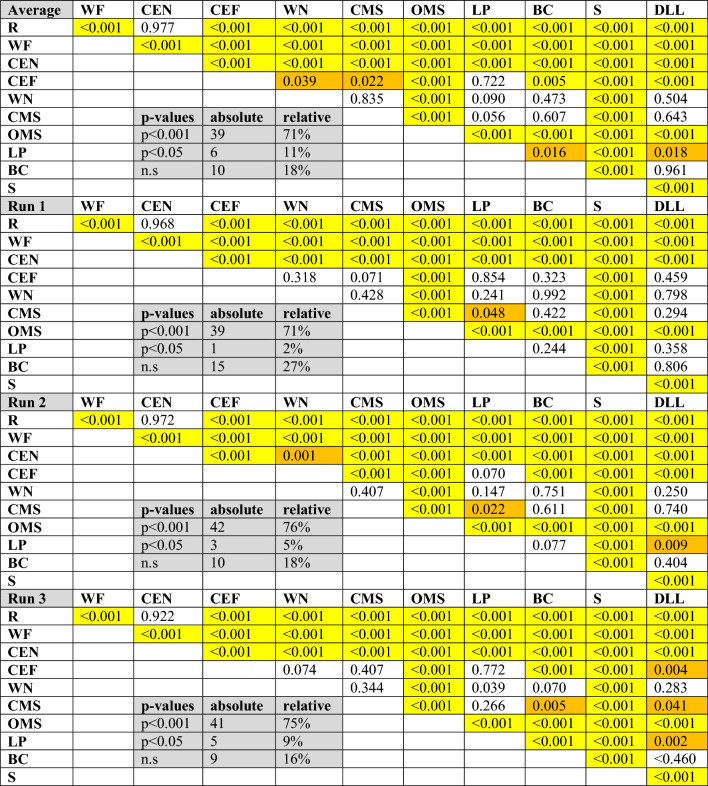
Discrimination between the facial muscle activity during the different facial exercises by the patterns of wireless facial sEMG recordings.

*R* at rest, *WF* wrinkling of the forehead, *CEN* closing the eyes normally, *CEF* closing the eyes forcefully, *WN* wrinkling of the nose, *CMS* closed mouth smiling, *OMS* open mouth smiling, *LP* lip puckering, *BC* blowing-out the cheeks, *S* snarling, *DLL* depressing lower lips, *n.s.* not significant.

The comparisons for the average of the 3 runs is shown as well as the results for each of the three runs separately. The inserted gray boxes contain a summary of the facial exercises that can be distinguished by the EMG activity patterns.

Highly significant differences marked in yellow (p < 0.001), and significant values in orange (0.001 < p < 0.05).

Focusing on averaged values of all runs, wrinkling of the forehead (frowning), normal (gentle) eye closure, and snarling were different from all other exercises in the EMG activation pattern (all p < 0.05). In general, depressing the lower lips, forced eye closure and wrinkling the nose were the most difficult to discriminate from other exercises in decreasing order. Depressing the lower lips was impossible to differentiate from nose wrinkling, smiling with closed mouth, and blowing the cheeks (p > 0.05). A forced eye closure could not be differentiated from lip puckering (p > 0.05). Nose wrinkling was similar to closed mouth smiling, lip puckering, blowing the cheeks, and depressing the lower lips (all p > 0.05). Finally, blowing cheeks could not be differentiated from depressing lower lips. With increasing repetition number, the discrimination from other exercises improved for depressing the lower lips.

### Differences between the summatory facial muscle activation between the three runs of facial movement exercises

**Figure 4:**
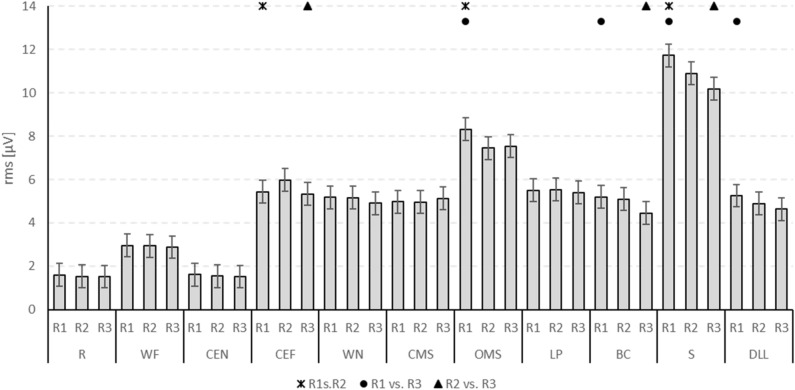
Electrode-independent facial muscle activation during specific facial exercises performed three times (three runs R1, R2, R3). The x-axis shows the different exercises. The y-axis shows the average values (± 95% confidence interval) of the root-mean-square (rms) of the sEMG amplitudes across all electrodes in µV. *R* at rest, *WF* wrinkling of the forehead, *CEN* closing the eyes normally, *CEF* closing the eyes forcefully, *WN* wrinkling of the nose, *CMS* closed mouth smiling, *OMS* open mouth smiling, *LP* lip puckering, *BC* blowing-out the cheeks, *S* snarling, *DLL* depressing lower lips. Asterisks (R1 vs. R2), dots (R1 vs. R3) and triangles (R2 vs. R3) indicate significant differences between the respective runs.

The overall facial muscle activation, i.e., the sum of the recordings of the entire electrode array, during each of the 11 exercises is shown in Fig. 4. The highest summatory facial muscle activation is seen during snarling, followed by smiling with open mouth. The lowest activation was observed during resting and closing the eyes normally. The electrode independent average activation decreased during the repetitions in most exercises.

## Discussion

The standard for high-resolution EMG to discriminate between facial exercises or emotional expressions in psychosocial and medical research is the use of standard gel electrode pairs on the skin^18^. Typically, 40 electrodes are placed on the face orientated along the topographical position of the underlying facial muscles^4^. The electrodes have to be placed in the direction of the muscle fibers and with constant inter-electrode distance. Reaching high accuracy in electrode placement to produce reliable results is time-consuming and requires a lot of experience. High-density sEMG for special settings even use up to 90 electrodes^19^. Such high-density recordings are so complex that they are not suitable for routine applications. An alternative to electrode-by-electrode placement is fixed geometric arrangement designed to match typical facial features, in a manner similar to electroencephalography (EEG) caps^8^. Placement of such electrode arrays does not mandate detailed knowledge of facial muscle anatomy nor a time-consuming process. Several recent studies have shown that the smart skin electrode array technology used in the present study is much easier and faster to apply^9–11^. What is more, when repeated sessions are required for the same individual, the arrays allow placement of a large number of electrodes at almost identical positions. Hence, the aim of the present study was to prove if whether the recordings of such a self-adhesive EMG foil mask are precise enough to reliably distinguish between defined facial movements.

Although we did not perform a head-to-head comparison to standard sEMG settings^5,6^, we assert that the values shown here for the discrimination of different facial exercises are sufficient for common psychosocial or medical research settings. It has already been shown that the sEMG arrays can be used to detect emotions^9^. Whether the adhesive sEMG masks are also reliable for the discrimination of imitated basic emotions that remains to be confirmed in future studies^7^. The present study has not yet made use of the second advantage of the sEMG masks: The wireless design does not restrict participants to sit upright in a chair. Demeco et al. used four wireless sEMG electrode pairs for facial muscle recordings in patients with facial palsy^20^. This allowed better quantification of a synchronously applied video movement analysis. Unfortunately, the source of the electrodes and the usability as medical device used in the aforementioned study is not described. It remains unclear if the wireless design in combination with lightweight of the films will allow more natural or at least unhindered facial movements. Therefore, a head-to-head comparison with other settings and an analysis of the movement would be needed. Recently, we have shown that it is possible to digitally remove electrodes from images and videos of participants performing facial expressions using machine learning algorithms^9,21^. We assume that similar ability would also be feasible (even easier) with the EMG masks, as they conceal less of the face (due to the lack of wires and minimal dimension of the electrodes). This would allow meaning automated image analysis of the facial surface synchronously to sEMG recording with the sEMG arrays to combine the strengths of both methods for an optimal discrimination of functional facial and emotional expressions^22–24^.

The present study has limitations. Using the video self-tutorial to demonstrate the facial movement tasks seems to be a very reliable observer-independent instruction technique but uses an imitation design to perform facial expressions^13^. A wireless application will allow more natural settings in future trials. Furthermore, the comparison between the three sessions showed that there is some variance in the performance of the exercises by the participants, as no feedback is implemented^18,25,26^. Hence, the problem in differentiating between different facial expressions may not only reflect on the presented electrode array system but on the subjects.

Depressing the lower lips, an exercise which might be performed infrequently in daily, showed the highest variability. The question of the optimum design, electrode positions, and the positioning of the DAU box in the EMG mask remains unanswered. Additional electrodes on the depressor anguli oris muscle and on the mentalis muscle would possibly help to achieve better discrimination between, for instance, the DLL and LP tasks. The depressor anguli oris muscle is important for DLL and the mentalis muscle for LP^27,28^. Furthermore, perhaps the difference between CEN and CEF would become clearer if the pars palpebralis of the orbicularis were also included into the recordings. Facialis midline muscles could also be better recorded. For instance, the procerus muscle is not yet included^29^. The procerus muscles is important for frowning and to expression emotional distress^29^. An advantage of the EMG foils is that they can, in principle, be printed in any shape for future trials. It will be important to print a mirror-inverted version of the mask to allow bilateral recordings.

As the discrimination from other exercises became better from session to session, one might conclude that it is advisable to take advantage of such a learning curve when using the present set of facial expression imitations in future trials. It can be assumed that trained users show a lower variability than untrained users. It is to be expected that an objective discrimination with the sEMG mask will then be even more precise^15^. It has already be shown that the used sEMG mask was more reliable than visual analysis when discriminating facial expressions between different sessions^15^. It has to be shown in future studies if this advantage holds true for repeated sessions at a greater distance, for instance, at intervals of months.

## Conclusions

Wireless high-resolution sEMG mask consisting of an adhesive electrode array film allowed a reliable discrimination of most standardized facial expressions in healthy adults. We recommend using the wireless adhesive multichannel sEMG system in psychosocial and medical research to also take advantage of the benefits of wireless use and the ease to attach them in settings with repetition over several days’ sessions.

### Supplementary Information

Supplementary Figures.

Supplementary Information.

## Data Availability

The original contributions presented in the study are included in the article/Supplementary material, further inquiries can be directed to the corresponding author.
