# Supplementary material for: Wireless high-resolution surface facial electromyography mask for discrimination of standardized facial expressions in healthy adults
Source: Sci Rep. 2024 Aug 20;14:19317. doi: 10.1038/s41598-024-70205-z (PMC11336214; doi:10.1038/s41598-024-70205-z)

Supplementary Figure S1-S12

Paul Funk et al. Wireless high-resolution surface facial electromyography analysis with adhesive electrode array foils for discrimination of standardized facial expressions in healthy adults: A prospective observational study

Legends

Supplementary Figure S1. Overview of the standardized facial movements (page 2)

Supplementary Figures S2-12. Average sEMG activation of all subjects of the different facial electrodes (channels) shown for each exercise separately (pages 3-13).
The upper graph always shows meant amplitude values of the sEMG (rms values in µV). The lower graph always shows relative values normalized to the maximum (separately per run). Statistically significant differences between runs are symbolized with dots (R1 vs. R2), rectangles (R1 vs. R3), and triangles (R2 vs. R3).

Abbreviations for the 11 exercises in sequence of presentation:

R	at rest
WF	wrinkling of the forehead
CEN	closing the eyes normally
CEF	closing the eyes forcefully
WN	wrinkling of the nose
CMS	closed mouth smiling
OMS	open mouth smiling
LP	lip puckering
BC	blowing-out the cheeks
S	snarling
DLL	depressing lower lips

Supplementary Figure S1

The standardized exercises

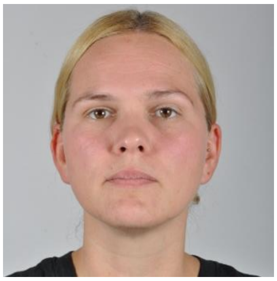

Face at rest

(R)

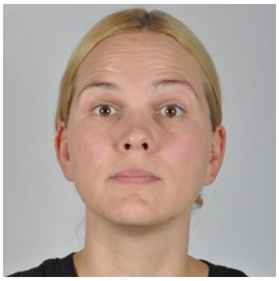

Wrinkling of the forehead

(WF)

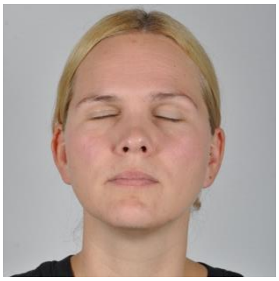

Closing the eyes normally

(CEN)

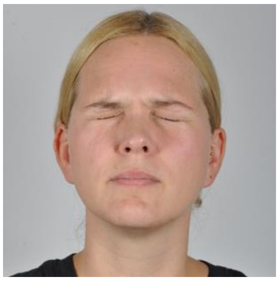

closing the eyes forcefully

(CEF)

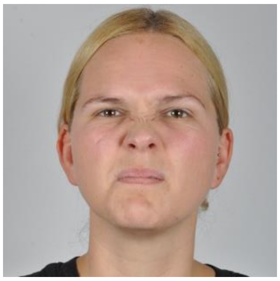

Wrinkling of the nose

(WN)

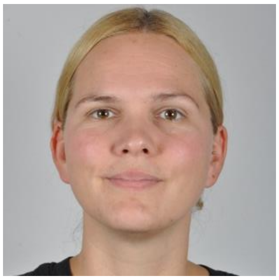

Closed mouth smiling

(CMS)

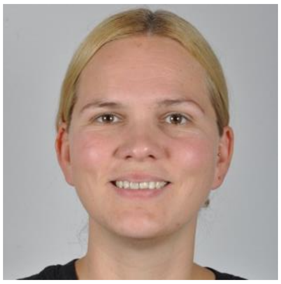

Open mouth smiling

(OMS)

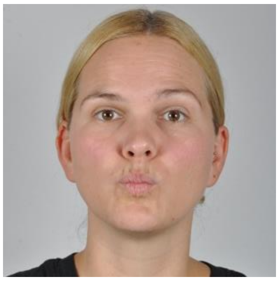

Lip puckering

(LP)

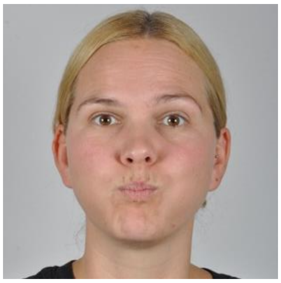

Blowing-out the cheeks

(BC)

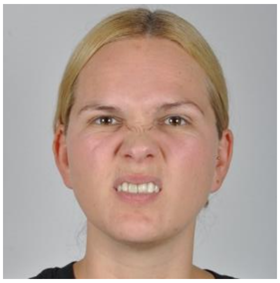

Snarling

(S)

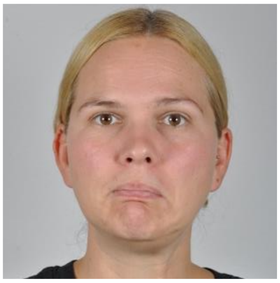

Depressing lower lips

(DLL)

Supplementary Figure S2

Exercise

At rest (R)

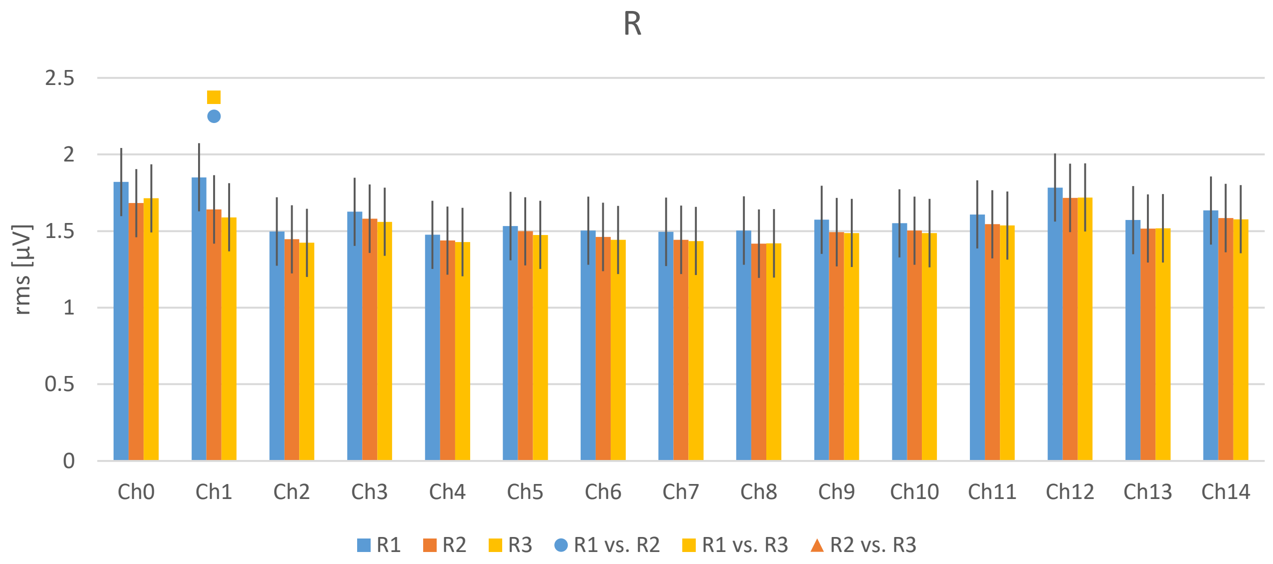

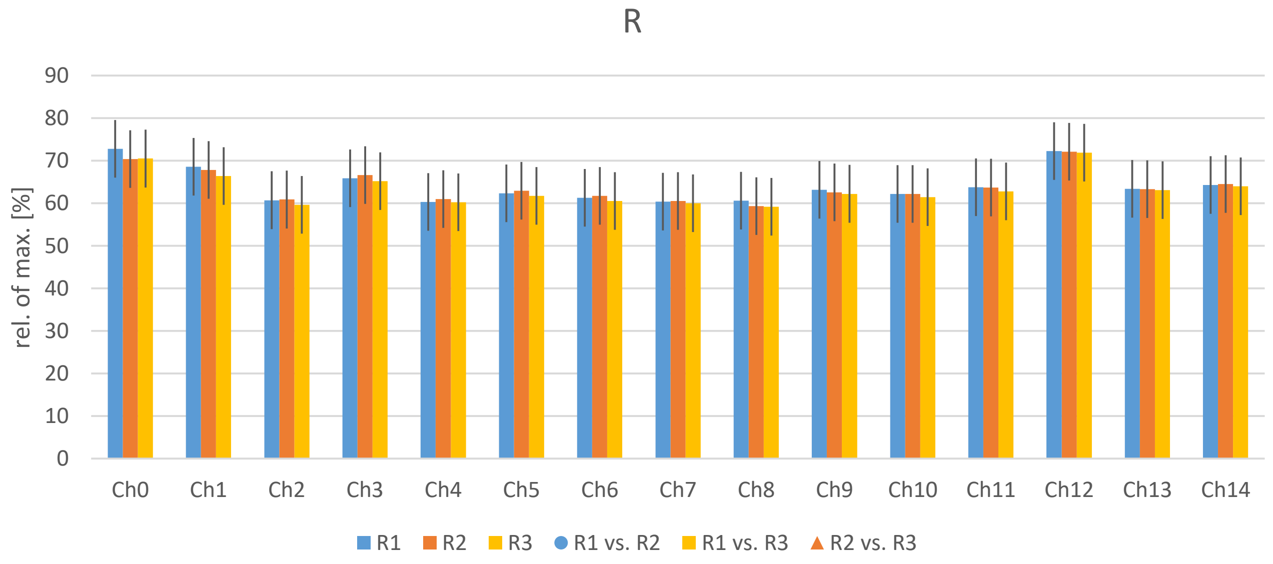

Supplementary Figure S3

Exercise
Wrinkling of the forehead (WF)

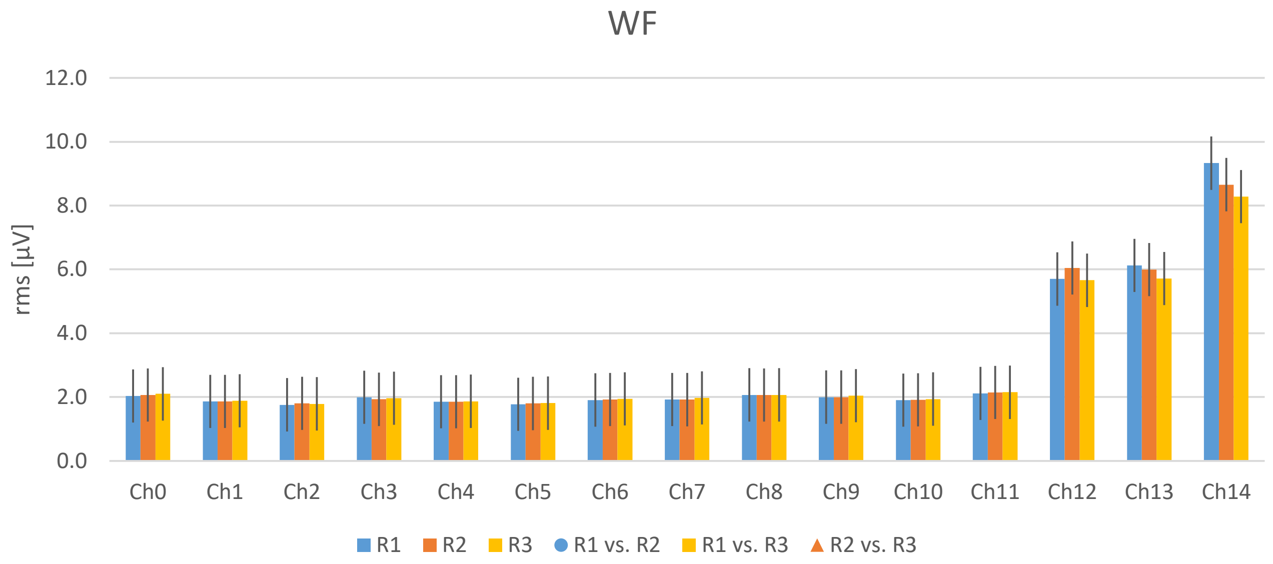

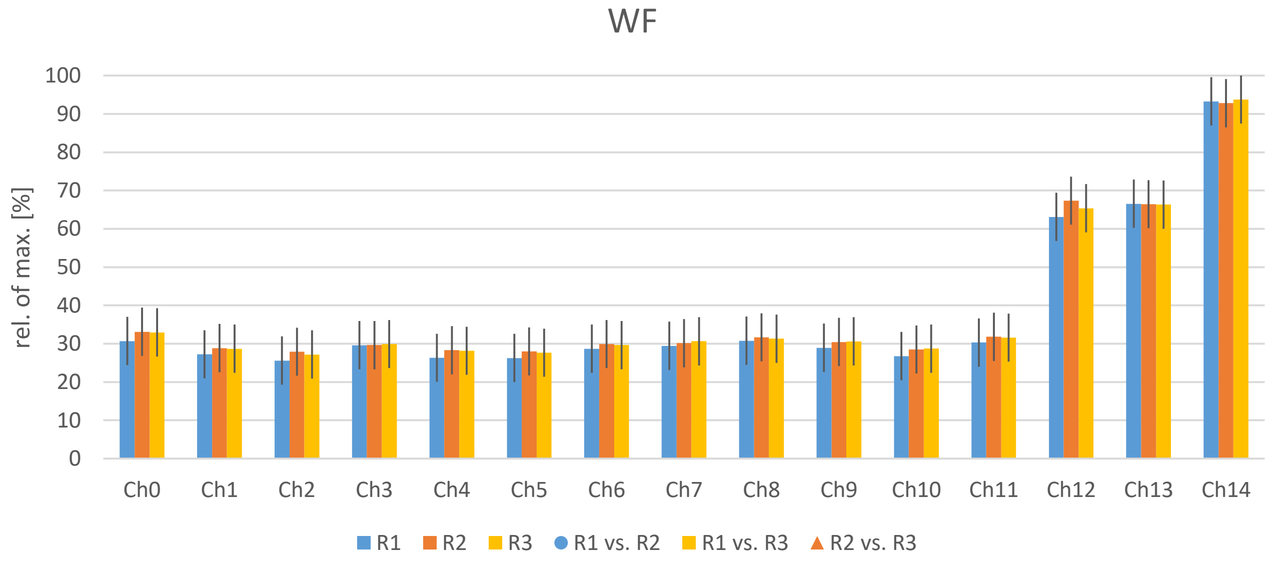

Supplementary Figure S4

Exercise
Closing the eyes normally (CEN)

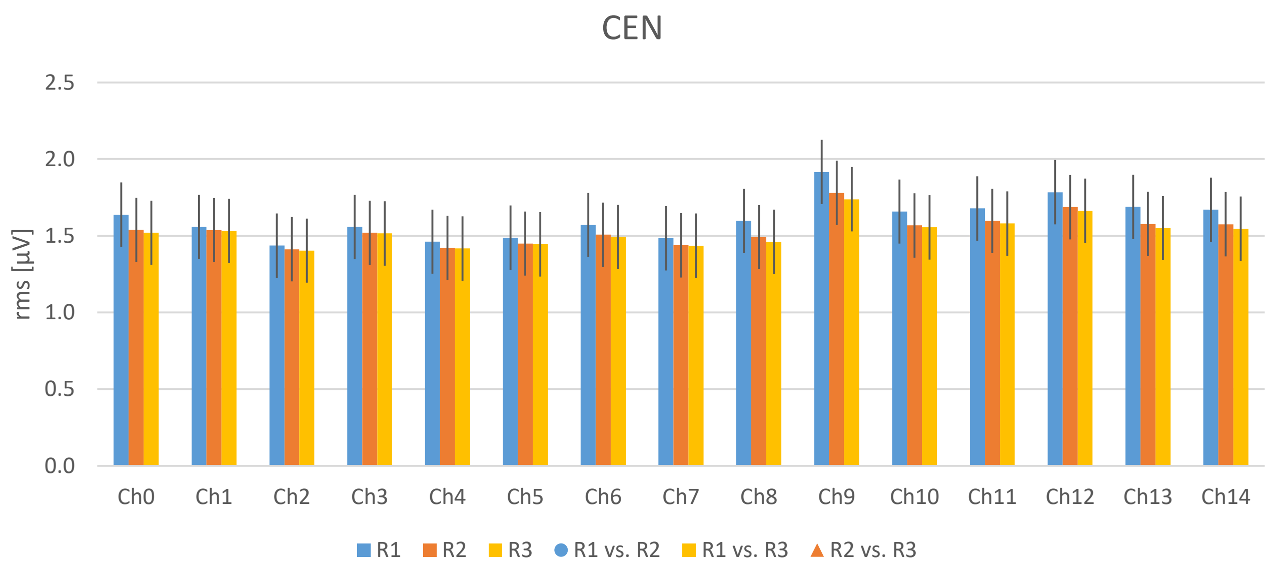

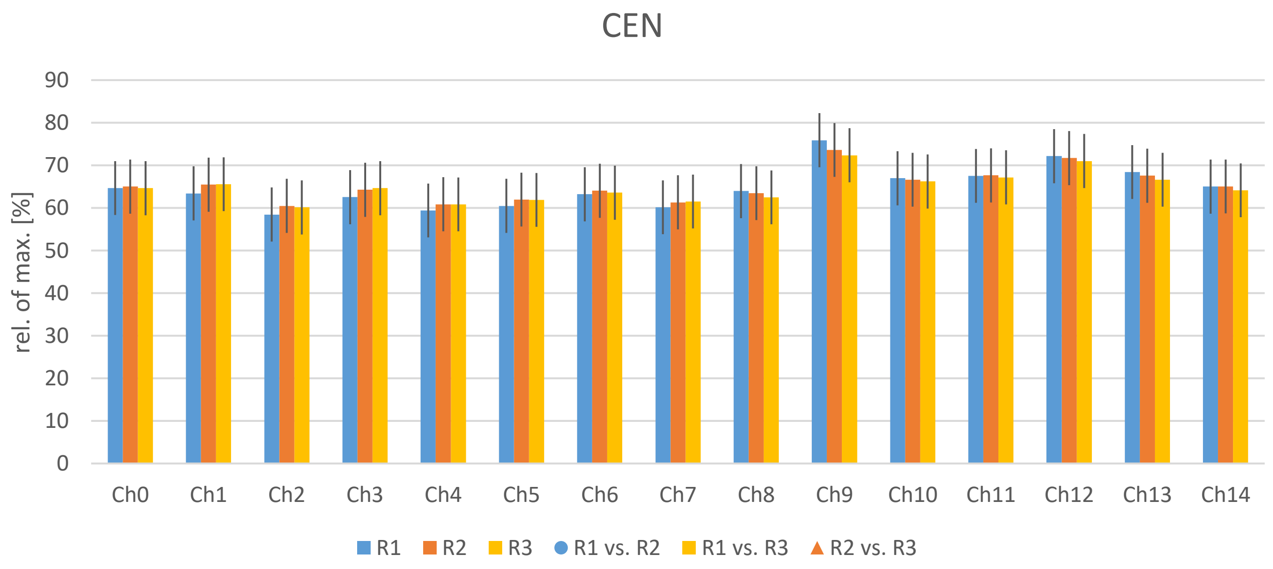

Supplementary Figure S5

Exercise
Closing the eyes forcefully (CEF)

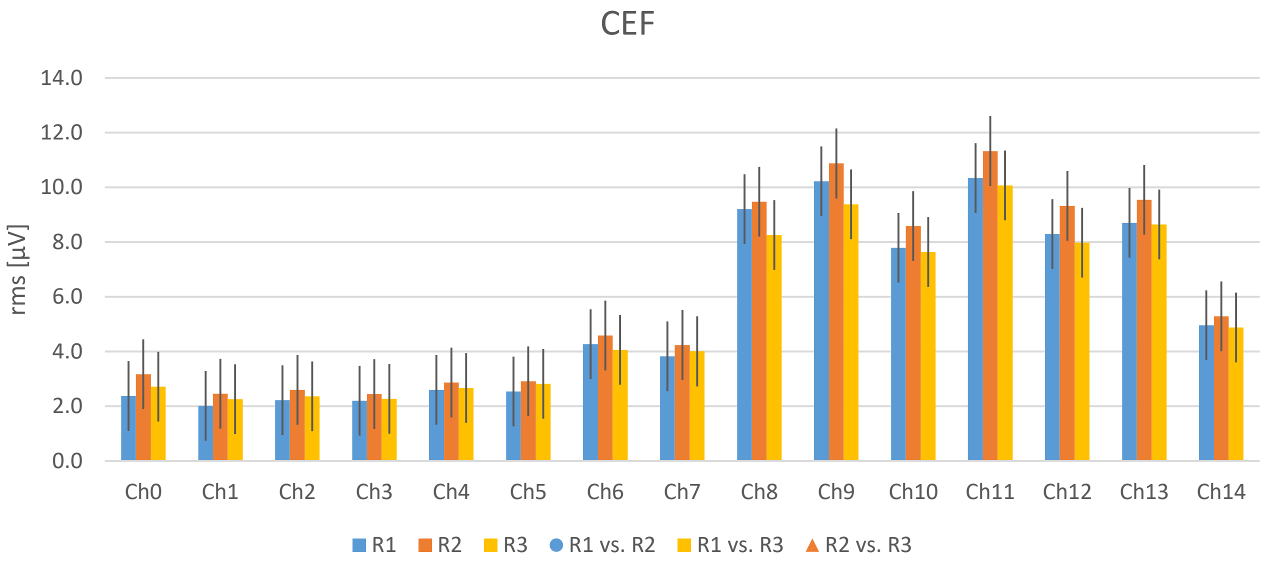

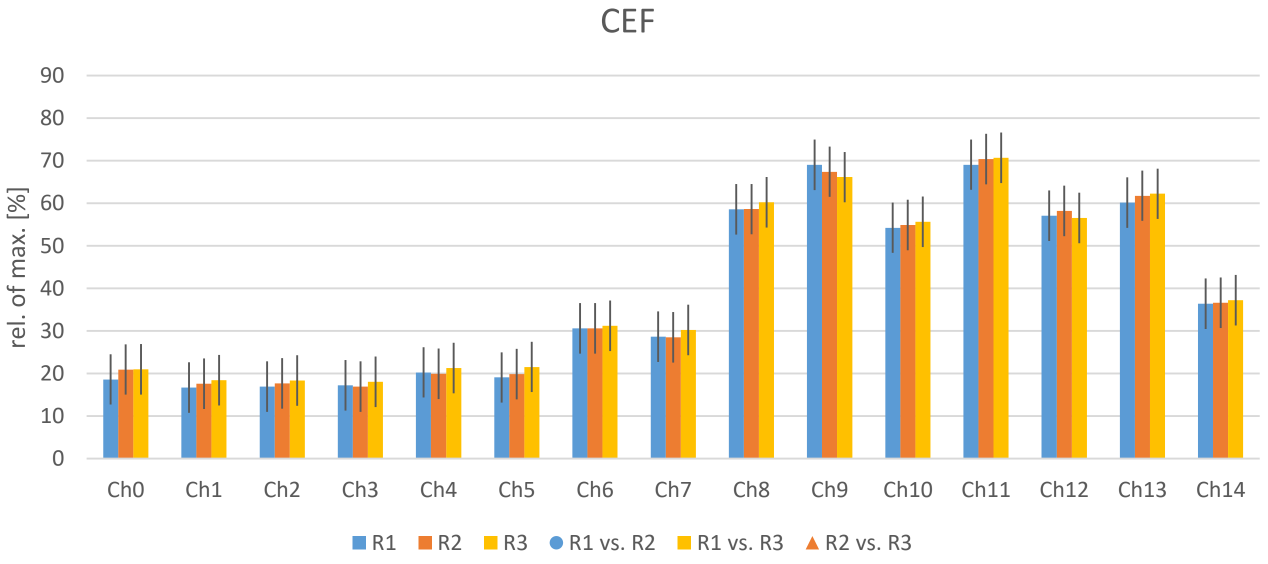

Supplementary Figure S6

Exercise
Wrinkling of the nose (WN)

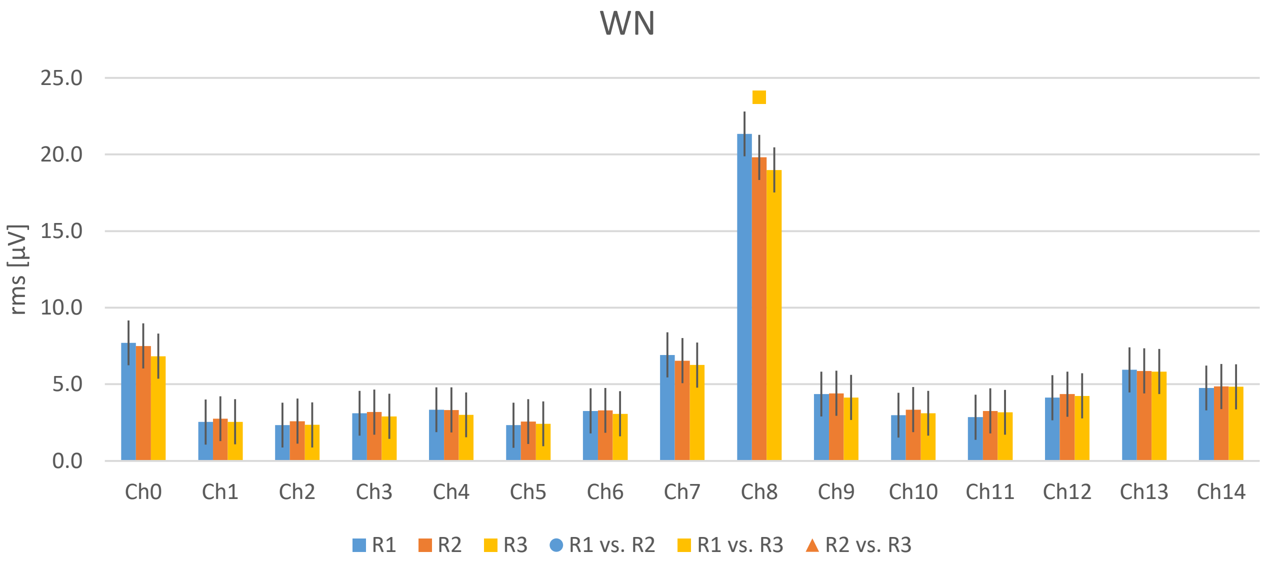

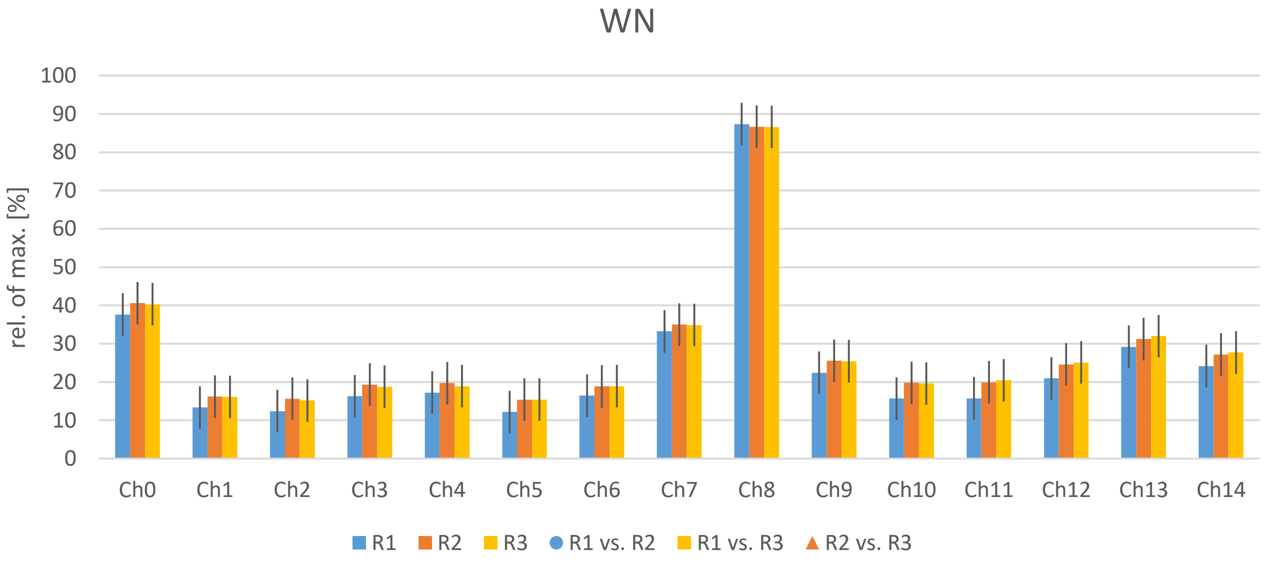

Supplementary Figure S7

Exercise
Closed mouth smiling (CMS)

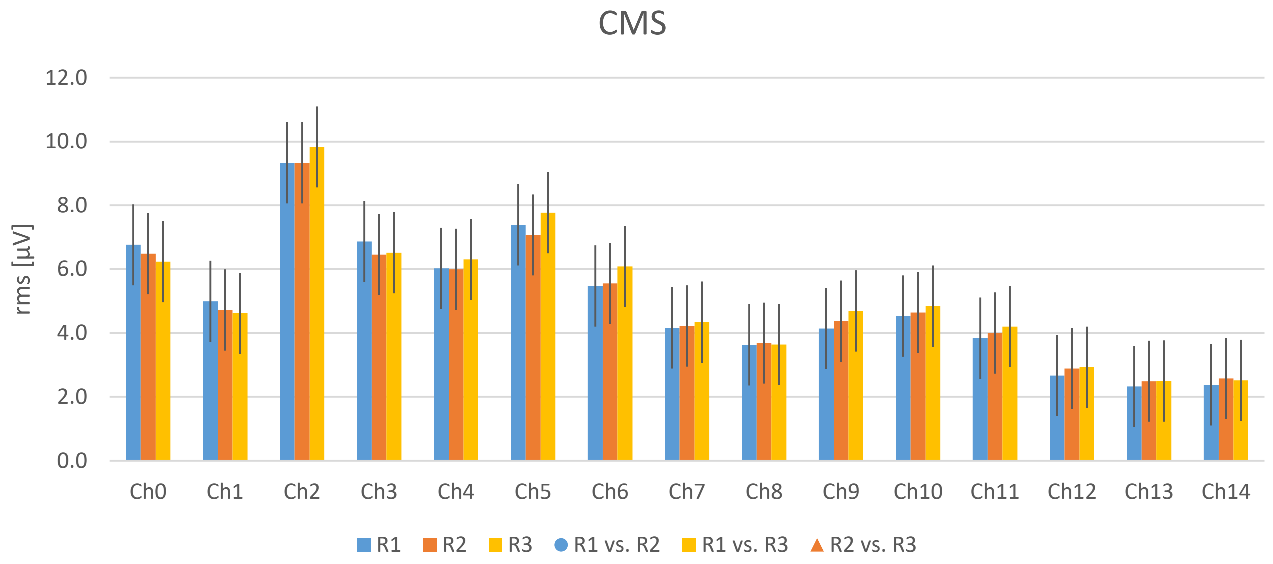

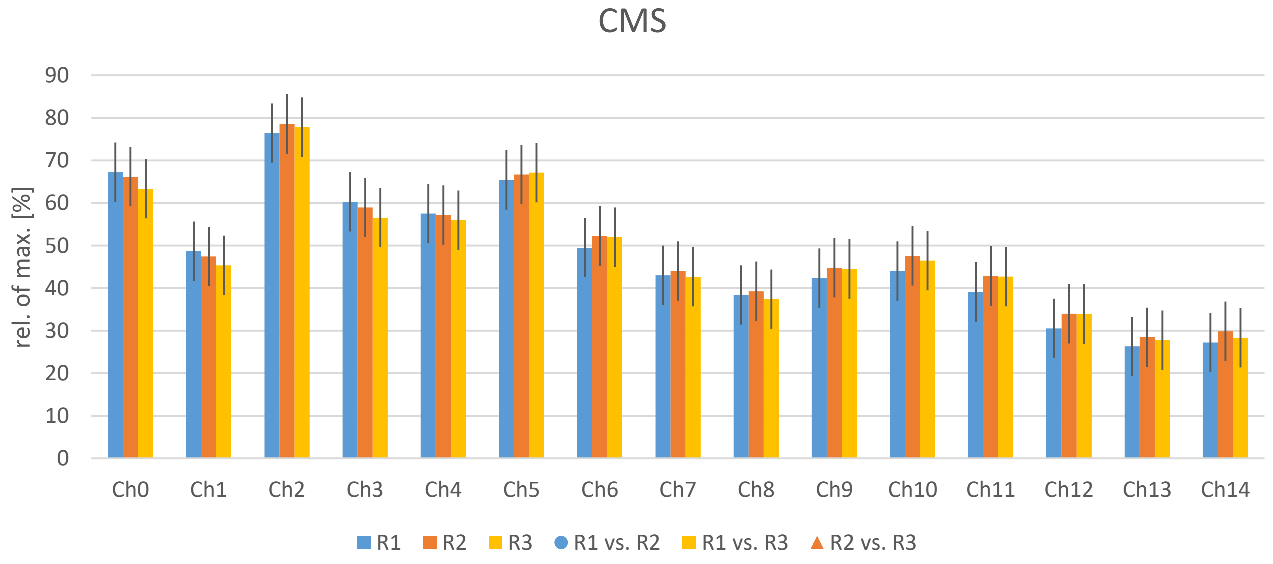

Supplementary Figure S8

Exercise
Open mouth smiling (OMS)

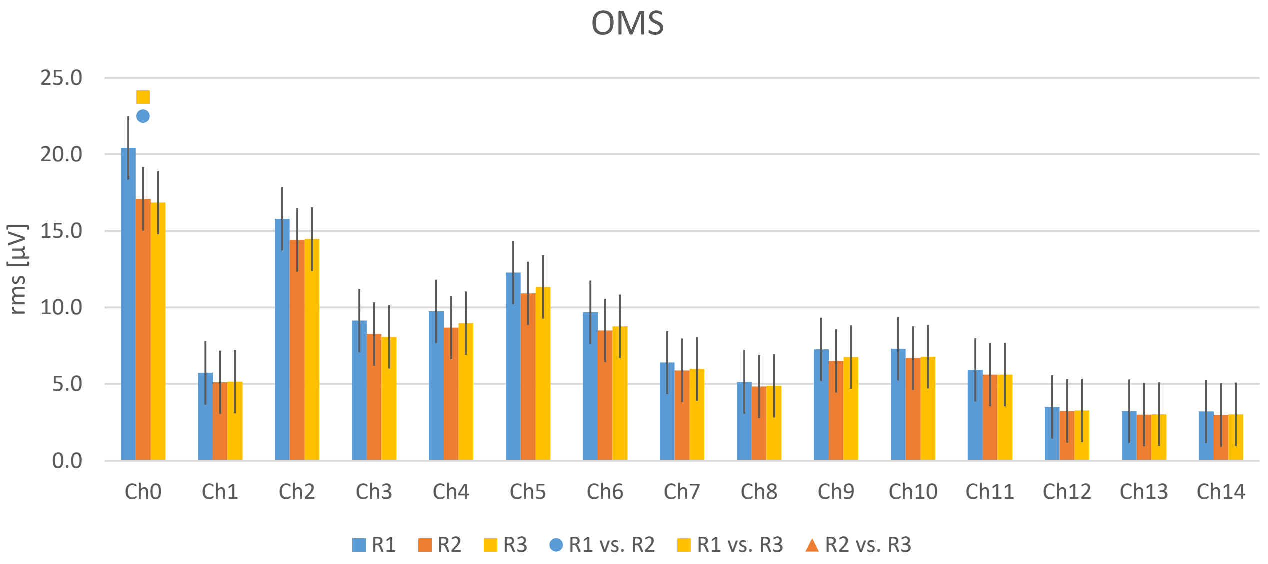

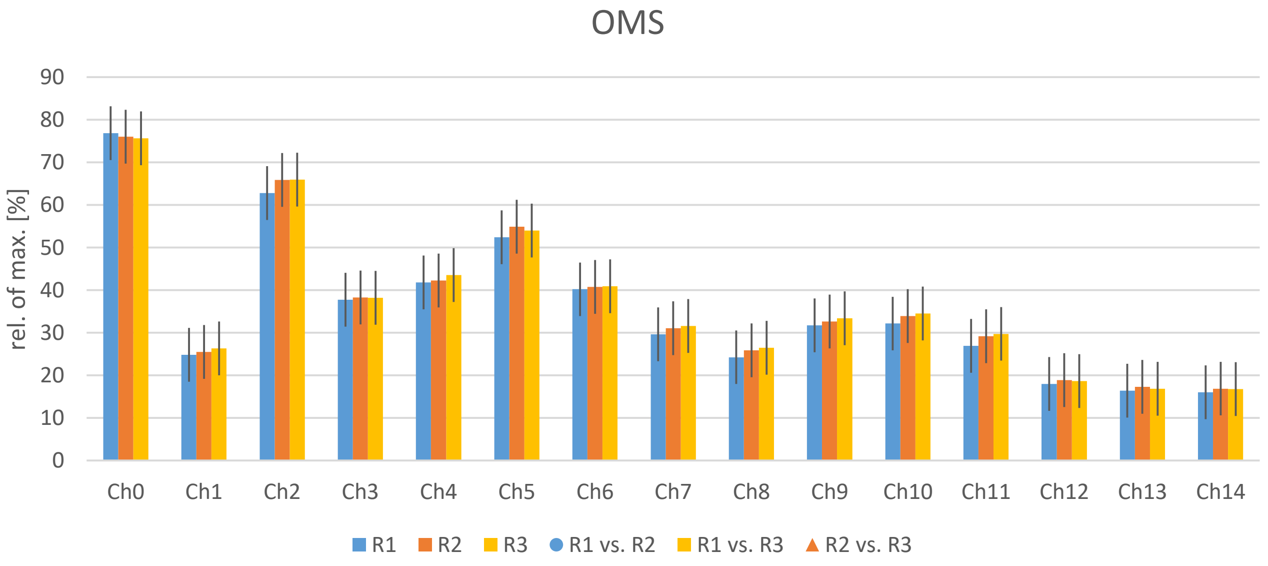

Supplementary Figure S9

Exercise
Lip puckering (LP)

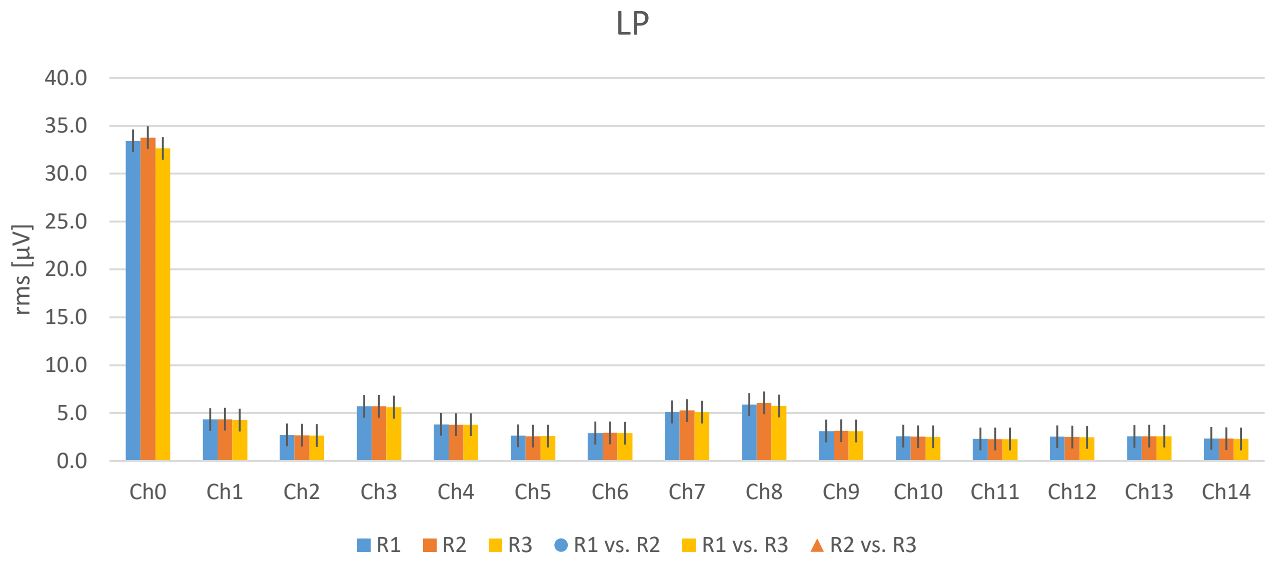

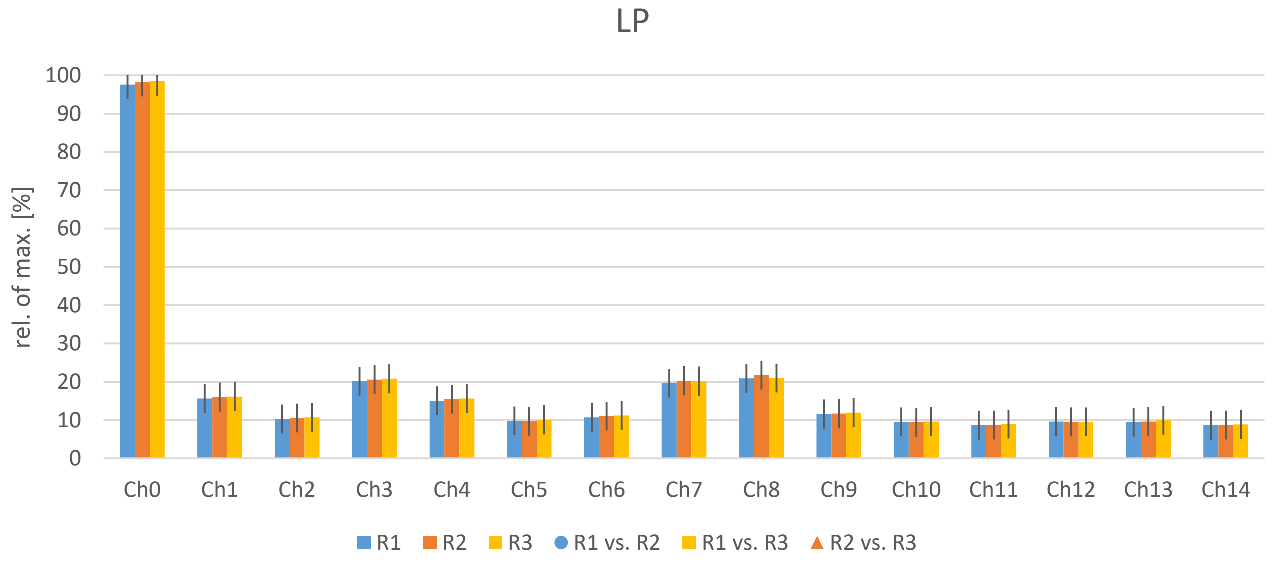

Supplementary Figure S10

Exercise
Blowing-out the cheeks (BC)

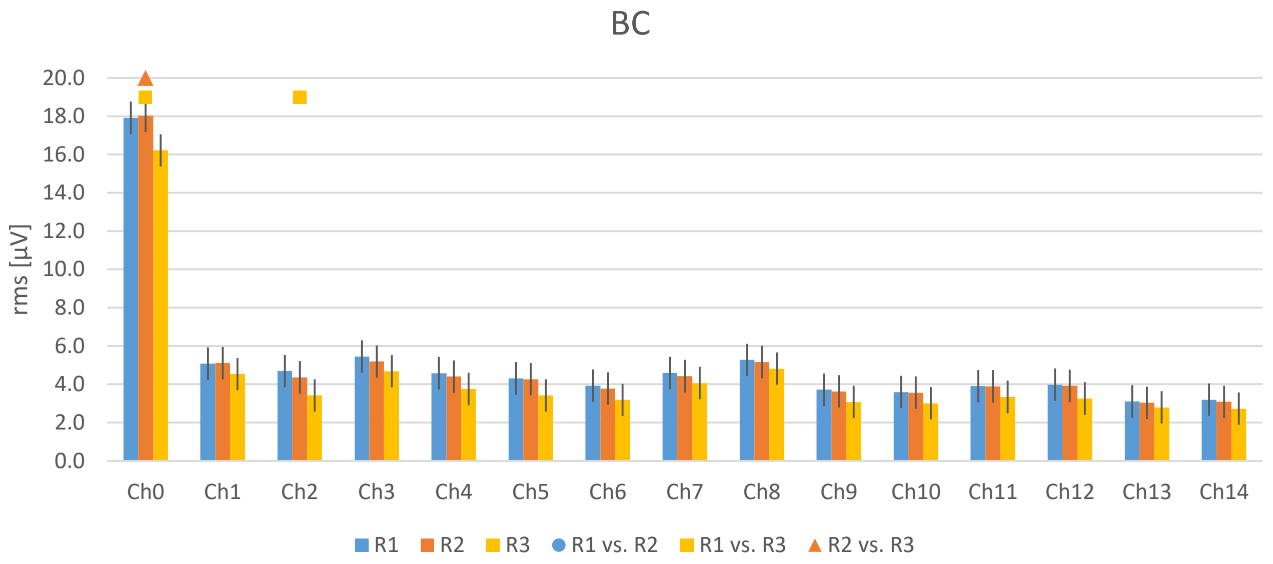

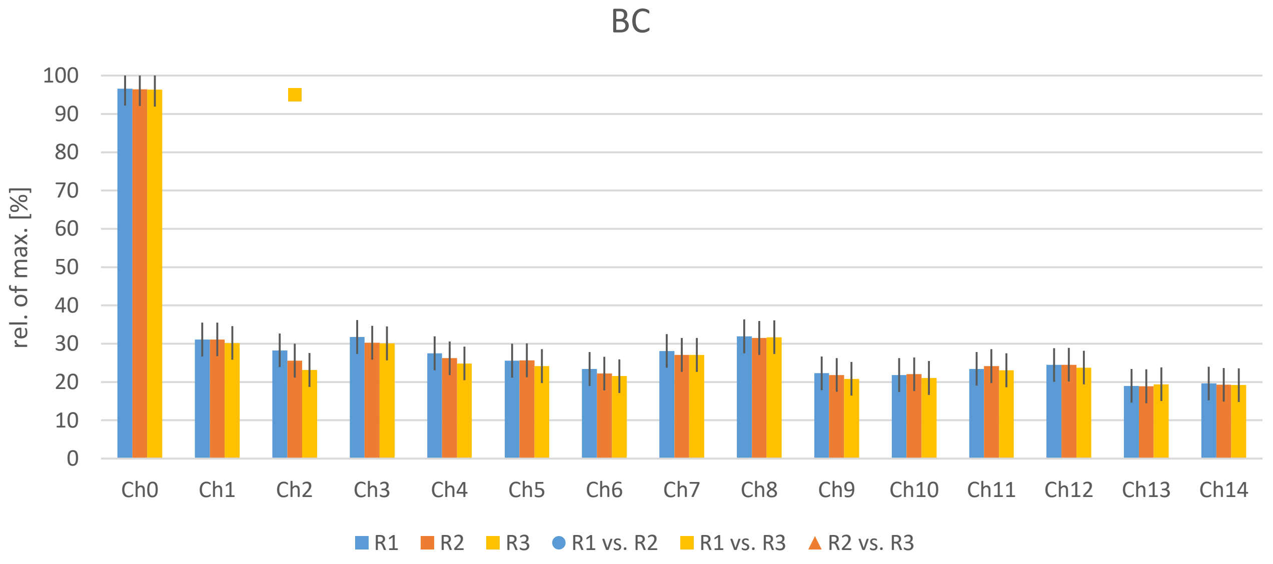

Supplementary Figure S11

Exercise
Snarling (S)

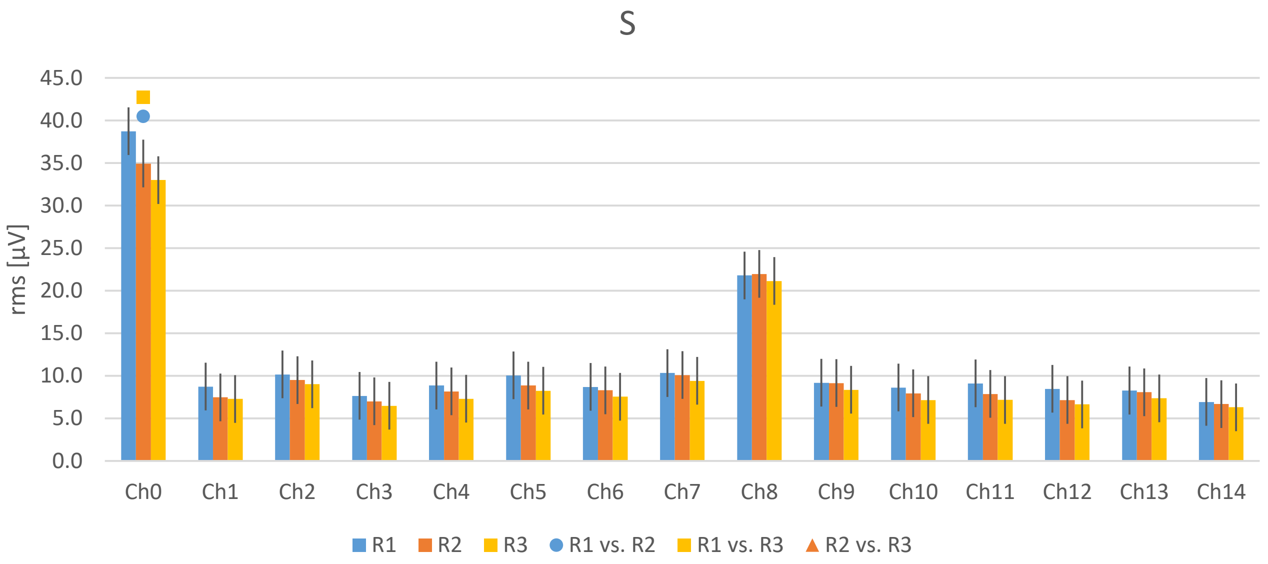

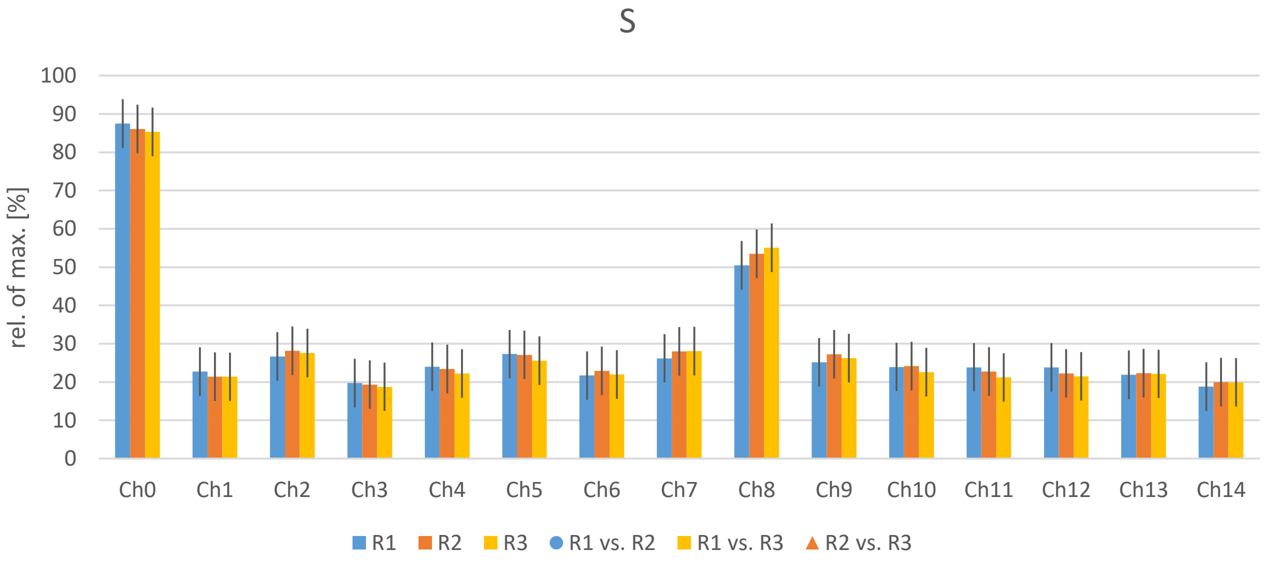

Supplementary Figure S12

Exercise
Depressing the lower lips (DLL)

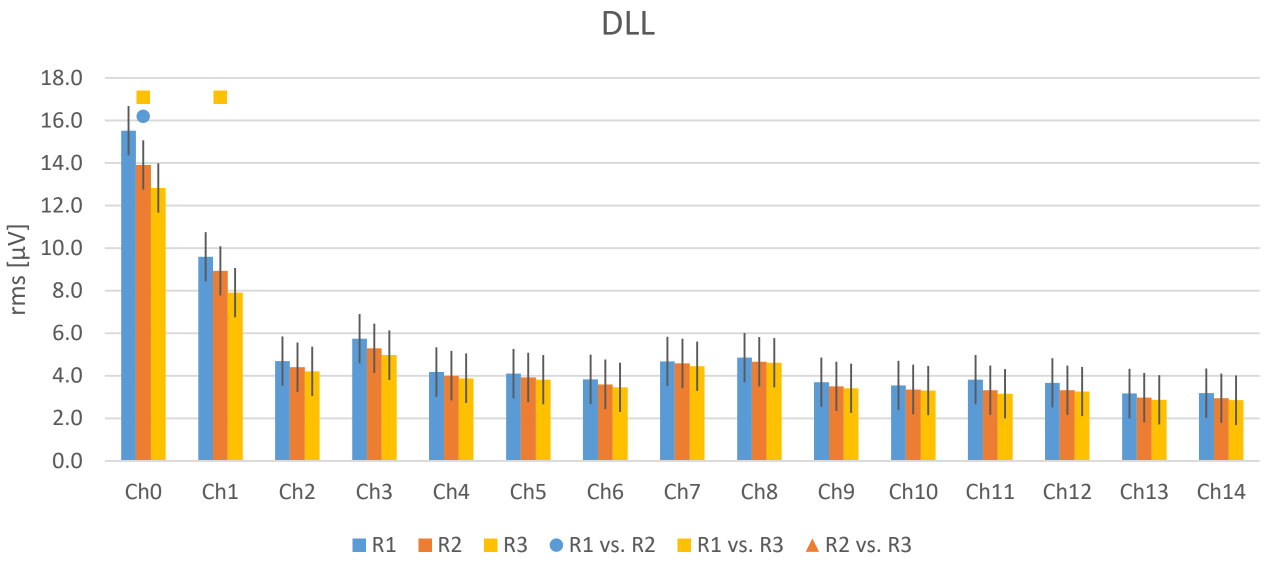

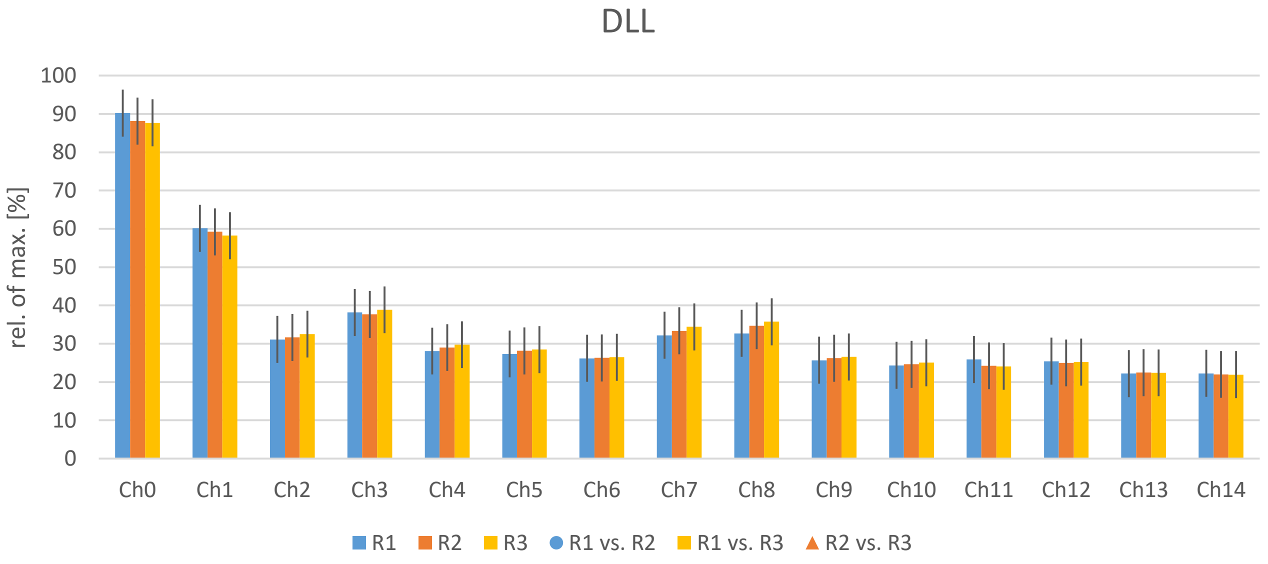

Supplement: Supplementary file 1 — Supplementary Figures. [file 41598_2024_70205_MOESM1_ESM.pdf]
